# Activation of RAS family members confers resistance to ROS1 targeting drugs

**DOI:** 10.18632/oncotarget.3311

**Published:** 2014-12-31

**Authors:** Marilisa Cargnelutti, Simona Corso, Margherita Pergolizzi, Laurence Mévellec, Dara L. Aisner, Rafal Dziadziuszko, Marileila Varella-Garcia, Paolo M. Comoglio, Robert C. Doebele, Jorge Vialard, Silvia Giordano

**Affiliations:** ^1^ Candiolo Cancer Institute - FPO, IRCCS, Torino, Italy; ^2^ Department of Oncology, University of Torino, Italy; ^3^ Janssen Research & Development, Janssen-Cilag, Val-de-Reuil, France; ^4^ Department of Pathology, University of Colorado School of Medicine, Aurora, CO, USA; ^5^ Medical University of Gdansk, Poland; ^6^ Department of Medicine, Division of Medical Oncology University of Colorado School of Medicine, Aurora, CO, USA; ^7^ Janssen Research & Development, a division of Janssen Pharmaceutica NV, Beerse, Belgium

**Keywords:** drug resistance, RAS, ROS1, lung cancer, targeted therapy

## Abstract

The *ROS1* tyrosine kinase is activated in lung cancer as a consequence of chromosomal rearrangement. Although high response rates and disease control have been observed in lung cancer patients bearing rearranged ROS1 tumors (ROS1+) treated with the kinase inhibitor crizotinib, many of these patients eventually relapse.

To identify mechanisms of resistance to ROS1 inhibitors we generated resistant cells from HCC78 lung cancer cells bearing the SLC34A2-ROS1 rearrangement. We found that activation of the RAS pathway in the HCC78 cell model, due to either KRAS/NRAS mutations or to KRAS amplification, rendered the cells resistant to ROS1 inhibition. These cells were cross-resistant to different ROS1 inhibitors, but sensitive to inhibitors of the RAS signaling pathway. Interestingly, we identified focal KRAS amplification in a biopsy of a tumor from a patient that had become resistant to crizotinib treatment.

Altogether our data suggest that the activation of members of the RAS family can confer resistance to ROS1 inhibitors. This has important clinical implications as: (i) RAS genetic alterations in ROS1+ primary tumors are likely negative predictors of efficacy for targeted drugs and (ii) this kind of resistance is unlikely to be overcome by the use of more specific or more potent ROS1 targeting drugs.

## INTRODUCTION

Lung cancer is the leading cause of cancer related death, with about 1.6 million cases diagnosed yearly worldwide, resulting in 1.4 million deaths [1]. Approximately 85% of lung cancer cases are non-small-cell lung cancers (NSCLC), most commonly adenocarcinomas or squamous-cell carcinomas. Early stage NSCLC can be treated with curative lung resection, whereas advanced and metastatic NSCLC have very poor prognoses, with a 5% 5-year survival rate [2].

The treatment and diagnosis of NSCLC has been revolutionized by the development of targeted agents for cancers harboring specific cancer promoting genetic alterations. Thus, routine genetic testing for somatic mutations/rearrangements from NSCLC biopsies is becoming the standard for providing optimal patient care. The identification of oncogenic mutations in receptor tyrosine kinases (RTK) such as *EGFR* [3, 4] and, less commonly, rearrangement of *ALK*, *RET* and *ROS1* in adenocarcinoma [5], has influenced treatment strategies by providing rationale for treatment with tyrosine kinase inhibitors (TKI) directed against these targets. This has also contributed to the approval of erlotinib for mutated *EGFR* tumors [6, 7] and crizotinib for *ALK* rearranged neoplasms [8].

Preclinical and clinical data indicate that tyrosine kinase inhibitors are generally effective only in subsets of patients bearing tumors of a defined genotype. From a genetic perspective, each organ-specific or histologic tumor type is a collection of many relatively rare tumors, carrying different genetic alterations and thus with a likelihood of responding to different targeted inhibitors. Therefore, a prerequisite for a successful targeted therapy is a precise molecular annotation of tumors which allows selection of patients that could benefit from that therapy. Even with optimal patient identification, a fraction of patients do not respond *ab initio* (primary resistance). Moreover, after an initial clinical response, cancer often recurs due to the development of drug resistance (secondary resistance) [9]. Therefore, the challenges associated with targeted therapies are to predict the mechanisms that lead to resistance and to find treatment strategies to circumvent these hurdles.

ROS1 is a receptor tyrosine kinase closely related to ALK and LTK [10, 11]. ROS1 oncogenic activation has been observed in patients with different tumor types such as glioblastoma, NSCLC, cholangiocarcinoma, gastric, ovarian and colon carcinoma, angiosarcoma and inflammatory myofibroblastic tumors (IMT) [12]. In all these cases, ROS1 activation is associated with inter-chromosomal translocations or intra-chromosomal deletions that result in chimeric genes, comprised of the intracellular portion of ROS1 fused to a variety of different partners [12, 13]. These genetic rearrangements lead to protein fusions that exhibit constitutive kinase activity and are associated with sensitivity to TKIs. The kinase inhibitor crizotinib, which has been shown to negatively affect proliferation of cells expressing ROS1 fusions [14], has demonstrated clinical efficacy in ROS1 fusion positive NSCLC and IMT patients [15; Ou SI, et al. Crizotinib therapy for patients with advanced ROS^1^-rearranged non-small cell lung cancer (NSCLC), WCLC 2013 Meeting, 2013]. As seen with other kinase inhibitors used in the treatment of solid malignancies, resistance to crizotinib has been recently reported in patients bearing tumors containing *ROS1* fusions. Resistance was attributed to either acquired mutations in the ROS1 kinase domain [16] or activation of the EGFR pathway [17].

This study describes the identification of additional novel mechanisms of resistance to ROS1 targeted drugs.

## RESULTS

### Establishment of cell lines resistant to ROS1 inhibitors

**FIG.1:**
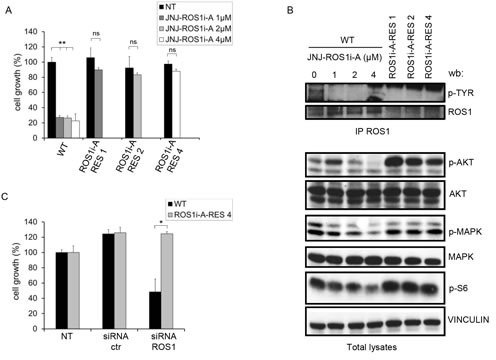
HCC78 cells resistant to the ROS1 inhibitor JNJ-ROS1i-A are not dependent on ROS1 for growth Parental cells were treated with the indicated concentrations of the inhibitor for 7 days. Resistant cells were kept in the presence of the drug concentrations to which they were made resistant. ns: not significant; **: significantly different from control (p< 0.01). B: Western blot analysis of HCC78 parental (WT) cells treated for 4 hours with JNJ-ROS1i-A with 0, 1, 2 or 4 μM and cells resistant to increasing concentrations of JNJ-ROS1i-A (ROS1i-A-RES 1, ROS1i-A-RES 2, ROS1i-A-RES 4). The blot was probed with the indicated antibodies. Vinculin was used as loading control. To evaluate ROS1 phosphorylation, cell lysates were immunoprecipitated with anti-ROS1 antibodies and the blot was probed with anti-phosphotyrosine antibodies. C: Growth rate of HCC78 cells (WT and ROS1i-A-RES 4) transfected with control or ROS1 specific siRNAs. Growth was evaluated after 96 hours of treatment. *: significantly different from control (p< 0.05).

The HCC78 lung cancer cell line is the prototype of “ROS1-addicted” cells, displaying the *SLC34A2-ROS1* gene rearrangement that leads to constitutive ROS1 kinase activation [11] and dependence on ROS1 for growth. We confirmed this dependence as treatment with a ROS1 inhibitor resulted in a strong impairment of cell viability and growth (Fig. 1A).

To determine potential mechanisms of acquired resistance to ROS1 kinase inhibitors, we used the specific inhibitor JNJ-ROS1i-A [Mevellec L, et al. Discovery of potent and selective RoS1 inhibitors with a unique DFG-out binding mode. 2014 AACR Annual Meeting. 2014]. This molecule inhibited the kinase activity of isolated recombinant ROS1 with an IC_50_ of approximately 30 nM. Growth of Ba/F3 cells engineered to express ROS1 and dependent on its kinase activity was inhibited at a similar concentration, as was ROS1 autophosphorylation in HCC78 cells. At the 1 μM concentration this compound inhibited less than 6% of kinases in a panel of 400. We treated HCC78 cells with increasing concentrations of JNJ-ROS1i-A for extended periods of time and thereby generated cell lines resistant to several concentrations of this inhibitor (1, 2, and 4 μM). The biological and biochemical properties of these resistant cells were then evaluated. The growth rate of the resistant cells was only slightly different from that of parental cells, both in the presence and in absence of the ROS1 inhibitor. A representative example is shown in Fig. 1A.

ROS1 phosphorylation was substantially decreased in these cells, while phosphorylation of AKT, MAPK and S6 kinase, components of downstream oncogenic signaling pathways, was maintained or increased (Fig. 1B). Interestingly, ROS1 protein and mRNA levels were substantially lower in resistant cells (Fig. 1B and Suppl. Fig. 1A) compared to the parental HCC78 cells. However, decreased transcription was not due to the loss of ROS1 gene copies (Suppl. Fig. 1B).

To demonstrate that the growth of these resistant cells was no longer dependent on ROS1, we transfected the parental and resistant HCC78 cells with ROS1 specific siRNAs (Suppl. Fig. 1C). As shown in Fig. 1C, growth of the parental cells was strongly impaired upon ROS1 silencing, while no substantial change was observed in resistant cells. These results suggest that resistance to the ROS1 inhibitor developed through a mechanism independent of the rearranged kinase.

### KRAS mutation confers resistance to ROS1 inhibitors

**FIG.2:**
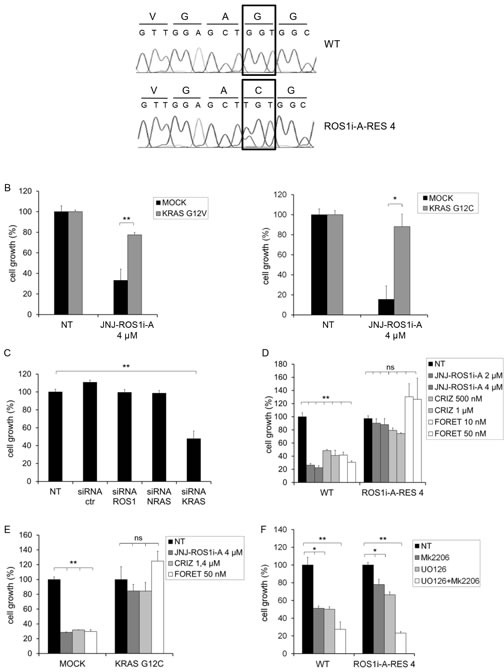
HCC78 cells resistant to the ROS1 inhibitor JNJ-ROS1i-A are KRAS addicted and cross-resistant to other ROS1 inhibitors A: DNA sequence analysis of HCC78 parental cells (WT) and cells resistant to the ROS1 inhibitor JNJ-ROS1i-A (ROS1i-A-RES 4). KRAS codon 12 of WT (upper panel) and ROS1i-A-RES 4 cells (lower panel) is indicated. B: Cell growth of HCC78 cells transduced with either an empty vector (MOCK) or constructs for expression of KRAS G12V (left panel) or KRAS G12C (right panel). The cells were untreated (NT) or treated with 4μM JNJ-ROS1i-A (6 days of treatment). *: significantly different from control (* p< 0.05; ** p< 0.01). C: Cell growth of ROS1i-A-RES 4 cells transfected with the indicated siRNAs and grown in the presence of JNJ-ROS1i-A 4μM. ROS1 and NRAS siRNAs were used as additional controls. D: Growth of parental (WT) or resistant (ROS1i-A-RES 4) HCC78 cells, treated with the indicated concentrations of JNJ-ROS1i-A, crizotinib or foretinib; ns: not significant; **: significantly different from control (p< 0.01). E: Growth of HCC78 WT cells transduced with empty vector (MOCK) or a KRAS G12C expression construct, treated with either 4 μM JNJ-ROS1i-A, 1.4 μM crizotinib or 50nM foretinib. Cell growth was measured after 6 days of treatment; ns: not significant; **: significantly different from control (p< 0.01). F: Growth of HCC78 cells (WT or ROS1i-A-RES 4), treated for 6 days with the MAPK inhibitor U0126 (10 μM), the AKT inhibitor MK2206 (3 μM), alone or in combination. *: significantly different from control (* p< 0.05; ** p< 0.01).

As described above, ROS1 inhibitor resistant HCC78 cells showed sustained activation of MAPK and AKT, despite the lower levels of ROS1 phosphorylation compared to parental cells. To determine the reason for this constitutive activation in the resistant cells, we looked for the presence of mutations in signal transducers that are frequently aberrantly activated in human tumors, such as *KRAS*, *NRAS*, *BRAF*, and *PIK3CA,* and are responsible for increased activation of MAPK and AKT. While no changes were found in the previously described mutational hot spots in *NRAS*, *BRAF* and *PIK3CA* (Suppl. Table 1), a mutation was detected in codon 12 (G12C) of *KRAS* (Fig. 2A). This mutation was not present in parental HCC78 cells. This is a well-known KRAS activating mutation, whose role as a negative predictor for the efficacy of tyrosine kinase inhibitors has been clearly shown in non-small cell lung cancer [18] and colorectal cancer [19].

To confirm that the presence of mutated *KRAS* could impair the response of HCC78 cells to ROS1 kinase inhibitors, we introduced *KRAS* cDNAs harboring either the G12C or the G12V mutation through viral transduction (Suppl. Fig. 2). As shown in Fig. 2B, cells expressing mutated *KRAS* were significantly less sensitive to JNJ-ROS1i-A. We also performed the inverse experiment, silencing KRAS in the resistant cells. Reduction of KRAS expression restored sensitivity of the resistant cells to JNJ-ROS1i-A (Fig. 2C and Suppl. Fig. 3). We also tested whether the presence of this mutation could render HCC78 cells resistant to other ROS1 inhibitors, such as crizotinib and foretinib [16, 20-22]. As shown in Fig. 2D and 2E, JNJ-ROS1i-A resistant and parental HCC78 cells expressing G12C KRAS were both insensitive to crizotinib and foretinib.

Because resistant cells display a strong activation of MAPK and AKT, we evaluated whether they were sensitive to inhibitors of these two kinases. Therefore, parental and ROS1 inhibitor resistant HCC78 cells were treated with U0126 and MK2206 (MAPK and AKT inhibitors, respectively), and their effects on growth were evaluated. As expected, resistant cells were sensitive to AKT and MAPK inhibitors (Fig. 2F), confirming dependence for growth on these pathways. Altogether these results point to KRAS activating mutations as a mechanism of resistance to ROS1 inhibitors.

### NRAS mutation confers resistance to ROS1 inhibitors

**FIG.3:**
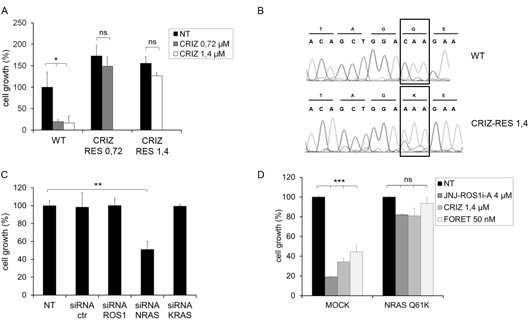
HCC78 cells resistant to crizotinib are addicted to NRAS A: Growth of parental (WT) and crizotinib resistant (CRIZ-RES 0.72 and CRIZ-RES 1.4) cells treated with the indicated concentration of crizotinib for 6 days. Resistant cells were kept in the presence of the drug concentration to which they were made resistant. ns: not significant; *: significantly different from control (p< 0.05). B: DNA sequence analysis of parental (WT) and crizotinib resistant (CRIZ-RES 1.4) HCC78 cells. NRAS codon 61 is highlighted. C: Cell growth of crizotinib resistant cells (CRIZ-RES 1.4) transfected with the indicated siRNAs and grown in the presence of crizotinib 1.4μM. ROS1 and KRAS siRNAs were used as additional controls. **: significantly different from control (p< 0.01). D: Growth of HCC78 cells transduced with empty vector (MOCK) or NRAS Q61K and subsequently untreated (NT) or treated for 6 days with 4 μM JNJ-ROS1i-A, 1.4 μM crizotinib or 50 nM foretinib. ns: nonsignificant; ***: significantly different from control (p< 0.001).

To further validate our observations on the role of RAS in mediating resistance to ROS1 TKIs, we generated HCC78 cells resistant to crizotinib, a ROS1 inhibitor that has demonstrated clinical activity in ROS1+ NSCLC and is currently undergoing clinical trials in ROS1+ patients (NCT00585195). We obtained cells resistant to 0.72 μM and 1.4 μM crizotinib (Fig. 3A). As was the case with JNJ-ROS1i-A (Suppl. Fig. 4A), crizotinib resistant cells displayed a reduction in ROS1 phosphorylation, decreased ROS1 expression (both at the protein and mRNA level) and sustained AKT and MAPK activation (Suppl. Fig. 4A, 4B). Sequencing did not reveal mutations in *KRAS, BRAF* or *PIK3CA* in these cells (Suppl. Fig. 4C and Suppl. Table 1). Interestingly, an NRAS activating mutation, Q61K, was detected in crizotinib resistant, but not parental HCC78 cells (Fig. 3B). Similarly to what was observed in JNJ-ROS1i-A resistant cells, sensitivity to crizotinib was restored upon NRAS silencing (Fig. 3C, Suppl. Fig. 3C). Moreover, expression of Q61K NRAS rendered parental HCC78 cells insensitive to crizotinib, JNJ-ROS1i-A and foretinib (Fig. 3D).

**FIG.4:**
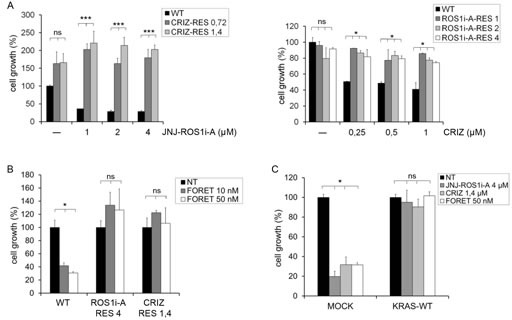
KRAS/NRAS activating mutations or KRAS overexpression render HCC78 cells resistant to ROS1 inhibitors A: Left panel: Growth of parental and crizotinib resistant HCC78 cells untreated or treated for 6 days with the indicated concentrations of JNJ-ROS1i-A. Right panel: Growth of parental and JNJ-ROS1i-A resistant HCC78 cells untreated or treated for 6 days with the indicated doses of crizotinib. ns: not significant; *: significantly different from control (p< 0.05); ***: significantly different from control (p< 0.001). B: Growth of parental (WT), JNJ-ROS1i-A resistant (ROS1i-A-RES 4) and crizotinib resistant (CRIZ-RES 1.4) HCC78 cells untreated (NT) or treated for 5 days with 10 and 50 nM foretinib. ns: not significant; *: significantly different from control (p< 0.05). C: Growth of HCC78 cells transduced with either an empty vector (MOCK) or a KRAS WT expression construct, untreated (NT) or treated with JNJ-ROS1i-A (4 μM), crizotinib (1.4 μM) or foretinib (50 nM). ns: not significant; *: significantly different from control (p< 0.05).

It has been previously shown that cells resistant to crizotinib as a consequence of mutations in the ROS1 kinase remain sensitive to the inhibitory activity of other ROS1 inhibitors, such as foretinib. In the present study, cells resistant to JNJ-ROS1i-A were cross-resistant to crizotinib and the crizotinib resistant cells were cross-resistant to JNJ-ROS1i-A (Fig. 4A). Moreover, both cell lines were also resistant to foretinib (Fig. 4B).

### Activation of the RAS pathway leads to down-regulation of SLC34A2-ROS1

**FIG.5:**
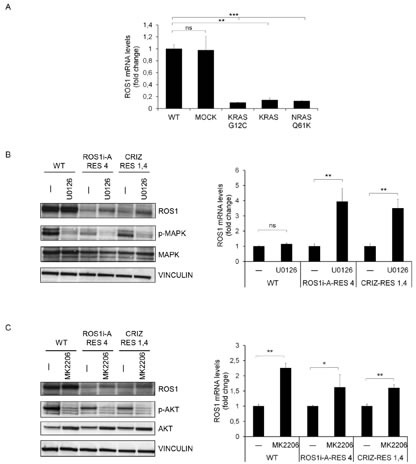
RAS pathway activation induces ROS1 down-regulation A: ROS1 mRNA levels in HCC78 cells transduced with empty vector (MOCK), KRAS G12C, KRAS or NRAS Q61K expression constructs. B: ROS1 protein (left panel) and mRNA (right panel) levels in HCC78 WT or ROS1 inhibitor resistant cells (ROS1i-A-RES 4 and CRIZ-RES 4), treated for 3 days with the MAPK inhibitor UO126. The left panel shows a WB of the treated cells, probed with the indicated antibodies. Vinculin was used as a loading control. The right panel shows ROS1 mRNA expression evaluated by qRT-PCT. ns: not significant; **: significantly different from control (p< 0.01). C: ROS1 protein (left panel) and mRNA (right panel) levels in HCC78 WT or ROS1 inhibitor resistant cells, treated for 3 days with the AKT inhibitor MK2206; significantly different from control (* p< 0.05; ** p< 0.01).

As described above, we observed a decrease of SLC34A2-ROS1 at the protein and mRNA level in both the JNJ-ROS1i-A and crizotinib resistant cells (Suppl. Fig. 1A, 4A). We hypothesized that this decrease could be a compensatory mechanism that prevents signal over-activation in cells with a constitutively active RAS pathway. We therefore assessed whether SLC34A2-ROS1 was also decreased in HCC78 cells transduced with viral constructs driving expression of constitutively active mutated KRAS or NRAS, or wt KRAS. As shown in Fig. 5A, cells with activated RAS pathway displayed a strong down-regulation of SLC34A2-ROS1 mRNA. To investigate whether RAS-dependent downstream signaling pathways were involved in SLC34A2-ROS1 down-regulation, we treated HCC78 cells resistant to either JNJ-ROS1i-A or crizotinib with MAPK or AKT inhibitors. Fig. 5B and 5C show that MAPK and AKT inhibition resulted in strong up-regulation of SLC34A2-ROS1, both at the mRNA and protein level, providing evidence for RAS pathway dependent regulation of SLC34A2-ROS1 expression.

### Elevated KRAS expression confers resistance to ROS1 inhibitors

As the role of RAS proteins in conferring resistance to tyrosine kinase inhibitors can be due not only to the presence of activating mutations, but also to amplification and elevated expression of RAS genes [23, 24], we wondered whether these mechanisms could also confer resistance to ROS1 inhibitors. HCC78 cells (bearing a normal KRAS copy number) were transduced with a wt KRAS expression construct (Suppl. Fig. 5A, 5B). These cells displayed a significantly reduced sensitivity to crizotinib, JNJ-ROS1i-A and foretinib (Fig. 4C).

Altogether these data show that genetic alterations (such as activating mutations and gene amplification) leading to constitutive activation of RAS family proteins can confer resistance to ROS1 inhibitors.

**FIG.6:**
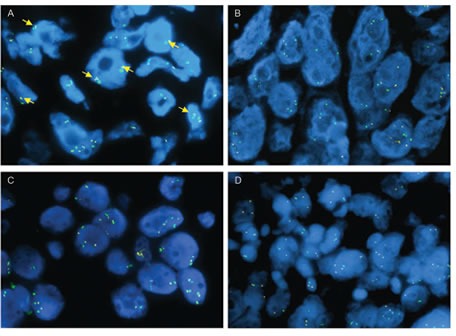
*KRAS* FISH analysis in ROS1+ tumor samples ROS1+ crizotinib-resistant patient tumor samples analyzed by FISH using *KRAS* (yellow) and CEP12 probes (green). Yellow arrows in panel A indicate mini-clusters corresponding to focal *KRAS* gene amplification in patient 1. Patient samples (2-4) in B-D were negative for KRAS gene amplification.

Based on our results, which demonstrate that RAS activation can mediate resistance to ROS1 inhibitors *in vitro*, we asked whether this pathway might also mediate resistance in ROS1+ NSCLC patients that develop acquired resistance to crizotinib. We therefore performed fluorescence *in situ* hybridization with probes binding to the genomic region of chromosome 12 harboring the *KRAS* gene on tumor samples from four patients with acquired resistance to crizotinib. One of these patients had focal *KRAS* amplification with a mean *KRAS* signal of 4.2 copies per cell and a mean *KRAS*/*CEP4* ratio of 2.1 (Fig. 6A). Unfortunately, a crizotinib pre-treatment sample was not available for comparative testing. The tumors from the remaining three patients did not display *KRAS* amplification (Fig. 6B-D). We also sequenced *KRAS*, *BRAF* and *NRAS* in two patient samples, but did not detect any mutations. Material from the remaining two patient samples was insufficient for analysis or failed quality control.

## DISCUSSION

ROS1 rearrangement was first described in 2003 in a glioblastoma cell line [25] and later identified in a non-small cell lung cancer (NSCLC) cell line [11]. Analysis of tumors from NSCLC patients showed that approximately 1% display rearrangements of the *ROS1* gene. *In vitro* studies confirmed that the rearranged ROS1 kinase is constitutively active and that it behaves as a tumor driver, since cells are addicted to its continuous activation. These observations implicated ROS1 as a candidate therapeutic target in NSCLC. ROS1 is closely related to the ALK kinase [26]; the tyrosine kinase domains of ALK and ROS1 have 77% amino acid identity within the ATP-binding sites. Because of this high level of homology, several ALK inhibitors are also active on ROS1 [27]. Preclinical studies demonstrating that the FDA-approved ALK inhibitor, crizotinib, also inhibits ROS1 provided rationale for its use in treatment of ROS1+ NSCLC patients [28]. In an ongoing Phase I study, crizotinib treatment resulted in a response rate of 60% (21 out of 35 patients) among patients with advanced *ROS1+* NSCLC [Ou SI, et al. Crizotinib therapy for patients with advanced ROS1-rearranged non-small cell lung cancer (NSCLC), WCLC 2013 Meeting, 2013]. However, as suggested by the clinical experience obtained with drugs targeting other receptor tyrosine kinases and by preclinical data, tumors treated with ROS1 inhibitors are likely to become resistant to therapy. Indeed, mechanisms of resistance to crizotinib, such as acquired ROS1 mutations leading to steric interference with drug binding [16] or EGFR pathway activation [17], have recently been described.

As knowledge of possible mechanisms of resistance could help in defining strategies aimed at either preventing or overcoming resistance, we generated ROS1+ HCC78 lung cancer cell resistant to ROS1 inhibitors. In this *in vitro* model we found that activation of the RAS pathway, due to either *KRAS* or *NRAS* mutations or to *KRAS* amplification, rendered the cells resistant to ROS1 targeting drugs. Activation of the RAS pathway has been described as a mechanism of primary or secondary resistance in colon cancer, where the presence of KRAS activating mutations in the primary tumor is a negative predictive factor of response to the anti-EGFR monoclonal antibodies cetuximab and panitumumab [29, 30]. Similar mutations and *KRAS* gene amplification have been shown to confer secondary resistance to anti-EGFR treatment in the same tumor type [31, 32]. Moreover, RAS pathway activation has been shown to predict primary resistance to imatinib in gastrointestinal stromal tumors [33] and to confer secondary resistance to MET inhibitors in MET-addicted cells [23]. However, the potential role of the RAS family in driving resistance to ROS1 inhibitors has not been reported so far. In this study we show that activation of the RAS pathway can confer both primary and secondary resistance in ROS1 addicted cells. Virus mediated expression of either wild type *KRAS* or constitutively active *KRAS* or *NRAS* in HCC78 cells resulted in decreased sensitivity to ROS1 inhibitors. Additionally, silencing of mutated RAS proteins in ROS1 inhibitor resistant HCC78 cells rescued sensitivity to the inhibitors. Interestingly, two different members of the RAS family were found mutated in the same cell line rendered resistant to two diverse inhibitors (NRAS in HCC78 cells resistant to the non-specific ROS1 inhibitor crizotinib and KRAS in HCC78 cell resistant to the specific inhibitor JNJ-ROS1i-A). This selective mutation/drug pairing (KRAS/JNJ-ROS1i-A and NRAS/crizotinib) was observed in resistant cell populations that were generated independently. This suggests that the drug might play a role in selecting cells present in the original population, harboring either *KRAS* or *NRAS* mutations and leading to the activation of one of these two pathways, which are similar but not identical. The differences in the spectrum of kinases inhibited by crizotinib and JNJ-ROS1i-A might influence this selective process.

To demonstrate that our findings *in vitro* are relevant in the clinical setting, we examined tumors from four ROS1+ NSCLC patients who relapsed upon crizotinib treatment for the presence of *KRAS*/*NRAS* mutations and *KRAS* amplification. Focal *KRAS* amplification was detected in one tumor, but we could not determine whether this event was present prior to crizotinib treatment or was acquired following treatment due to the absence of a pre-treatment sample.

Interestingly, we observed a decrease of *ROS1* protein in the inhibitor resistant cells. This does not seem to be restricted to RAS-mediated resistance as it has also been found in HCC78 cells resistant to crizotinib due to activation of the EGFR pathway [17]. Thus, down-regulation of the *ROS1* oncogene seems to be a common feature of resistance resulting from bypass signaling and is possibly due to a potentially detrimental effect of excessive signaling in these cells.

As previously mentioned, one of the major goals in the field of targeted therapy is to identify means to overcome resistance to treatment. It has been shown previously that foretinib, a potent inhibitor of oncogenic ROS1 fusion proteins [22], retains activity on the crizotinib resistant G2032R *ROS1* mutation. However, in our study foretinib was ineffective when resistance was due to the presence of activating *KRAS*/*NRAS* mutations or *KRAS* amplification. This can be explained by constitutive activation of the RAS pathway downstream of ROS1 that renders inhibition of this tyrosine kinase irrelevant. Concerning treatment, ROS1 resistant patients displaying RAS pathway activation are unlikely to be responsive to more potent and more specific new generation ROS1 inhibitors. As expected and confirmed in this study, these resistant cells are instead sensitive to inhibitors of the RAS pathway. Therapeutic options to efficiently target RAS are currently not available, but interesting opportunities have been recently reported [34]. One of these new drugs, indeed, targets the KRAS G12C mutation that confers resistance to JNJ-ROS1i-A [35].

The identification of RAS activation as a mechanism of resistance to ROS1 targeted therapies has some important clinical implications: (i) the identification of RAS genetic alterations in ROS1+ primary tumors would likely be a negative predictor of efficacy of targeted drugs; (ii) the post treatment identification of the same alterations would discourage treatment with new generation ROS1 inhibitors; (iii) the appearance of RAS mutations could be monitored in the plasma through the analysis of circulating DNA [31].

## METHODS

### Cell lines and reagents

HCC78 cells were purchased from the Deutsche Sammlung von Mikroorganismen und Zellkulturen (DSMZ, Braunschweig, Germany) cell bank and were cultured in RPMI medium 1640 (Sigma-Aldrich, St Louis, MO, USA) supplemented with 10% fetal bovine serum, 100 units/mL penicillin, and 100 μg/mL streptomycin. 293T cells were obtained from ATCC. The genetic identity of the cell lines was confirmed by short tandem repeat profiling (Cell ID, Promega, Madison, WI, USA), which was last repeated in March 2014.

Crizotinib and foretinib were purchased from Sequoia Research Products (Pangbourne, UK). MAPK inhibitor (U0126) was purchased from Promega and the AKT inhibitor (Mk2206) from Merck (Whitehouse Station, NJ, USA). The ROS1 inhibitor JNJ-ROS1i-A was provided by Janssen Pharmaceutica NV; Limited samples of compound JNJ-ROS1i-A are available for academic research from Janssen Pharmaceutica NV, and, subject to potential third party rights, can be made available to qualified researchers for their own academic research use, after the signature of a Material Transfer Agreement with Janssen Pharmaceutica NV detailing the terms and conditions for such research use. There are no additional restrictions from Janssen applicable to the above referred research use.

### Generation of HCC78 cells resistant to JNJ-ROS1i-A or crizotinib

HCC78 cells were exposed to increasing concentrations of JNJ-ROS1i-A or crizotinib, beginning with concentrations that resulted in reduction of culture growth by approximately 50 to 70%. The concentration was progressively increased every 15 to 30 days during a six month period, with changes in concentration taking place when the cells regained a growth rate similar to that of untreated cells. ROS1i-A-RES 1, 2, and 4 correspond to distinct HCC78 cell populations resistant to different concentrations (in μM) of JNJ-ROS1i-A; CRIZ-RES 0.72 and CRIZ-RES 1.4 indicate HCC78 cell populations resistant to different concentrations (in μM) of crizotinib. All the established resistant cell lines were maintained in culture in the presence of the inhibitor at the final concentration that was used to generate resistance.

### Patient samples

Tumor samples were tested following informed written consent from each patient. Patient details are provided in Suppl. Table 3.

### mRNA and gDNA analysis

mRNA and gDNA analyses were performed with standard techniques.

mRNA (500ng) extracted using miRNeasy Mini Kit (Qiagen, Venlo, Netherlands), was reverse transcribed into cDNA using the Multiscribe MuLV retrotranscriptase and random primers; cDNA (250ng) was amplified by Real-time qPCR using the Power SYBR Green PCR Master Mix, according to the manufacturer's protocol (Applied Biosystem, Foster City, CA, USA). Real-time qPCR was performed by using the following primers:

hROS1 Fw: 5′-GCCTTATCCAGCTCATTCCA-3′;
hROS1 Rw: 5′-AGGTCTTTGGTCGGGTTCTT-3′;
hACTIN Fw: 5′-GGAGGAGCTGGAAGCAGCC-3′;
hACTIN Rw: 5′-GCTGTGCTACGTCGCCCTG-3′;
hGAPDH Fw: 5′-GAAGGTGAAGGTCGGAGTC-3′;
hGAPDH Rw: 5′-GAAGATGGTGATGGGATTTC-3′;

Genomic DNA was extracted using the genomic DNA purification mini Kit (Qiagen) and amplified using Power SYBR Green PCR Master Mix (Applied Biosystems) by real-time quantitative PCR using ABI Prism 7900HT (Applied Biosystems). Specific primers were designed using Primer3 web tool (*):

genROS1 Fw: 5′-GTCCTCTAGGCTCCCAGGAAT
genROS1 Rw: 5′-CTTGCCAGAAGGGCAGTAAG
genKRAS Fw: 5′-GGGAGGGCTTTCTTTGTGTA
genKRAS Rw: 5′-TCCTGAGCCTGTTTTGTGTCT
STS6 Fw: 5′-CCTTCAAGAGAAAGACGACAG
STS6 Rw: 5′-AGGACTTATAAAAGGCAAGGG

Mutational analysis was performed via PCR amplification of 1 ng/μL genomic DNA using 0.25 mmol/L deoxynucleotide triphosphates, 1 μmol/L each of the forward and reverse primers (listed in Suppl. Table 2), 6% DMSO, 1x PCR reaction buffer, 0.05 unit/μL AmpliTaq Golden (Promega). PCR products were purified using AMPure (Agencourt Bioscience Corp., Beckman Coulter S.p.A, Milan, Italy) according to manufacturer procedures and analyzed on a 3730 DNA Analyzer, ABI capillary electrophoresis system (Applied Biosystems).

The SNaPshot assay for evaluation of multiple oncogenic mutations in *BRAF*, *KRAS*, *NRAS*, and other tumor-related genes was conducted by amplification using 13 multiplexed PCR reactions followed by single nucleotide base extension reactions. The products were separated by capillary electrophoresis and analyzed using GeneMapper 4.0 as has been previously described [36].

### Western blot analysis

Whole-protein extracts were prepared using LB buffer (½ vol. H_2_O, ¼ vol. Tris HCl pH 6.8, ¼ vol. SDS 10%) and quantified using the BCA Protein Assay kit (Pierce, Rockford, lL, USA). Protein extracts (60μg) were subjected to 8% SDS-PAGE, immunoblotted and analyzed using chemiluminescence ECL Western Blotting (Promega). The inhibitors JNJ-ROS1i-A and crizotinib were added 4 hours before cell lysis, where indicated. The following antibodies were used: anti-ROS (C-20, Santa Cruz Biotechnology, Santa Cruz, CA, USA); anti–phospho-AKT (Ser^473^), anti-AKT, anti–phospho-p44/42 MAPK, anti-p44/42 MAPK and anti pS6 Ribosomal Protein (Cell Signalling, Beverly, MA, USA); anti-KRAS and Vinculin (1931) (Sigma-Aldrich). Secondary IgG HRP-Peroxidase antibodies were obtained from Amersham (Uppsala, Sweden).

For immunoprecipitations, cells were lysed with EB buffer (20 mM Tris-HCl at pH 7.4, 5 mM EDTA, 150 mM sodium chloride, 10% glycerol and 1% Triton X-100) in the presence of 1 μg ml^−1^ leupeptin, 3 μg ml^−1^aprotinin, 1 μg ml^−1^pepstatin, 2 mM phenylmethylsulphonyl fluoride and 1 mM sodium orthovanadate. After immunoprecipitation with the anti-ROS1 Ab (C-20), high-stringency washes were performed. Phosphotyrosine was detected with the anti-phosphotyrosine clone 4G10 mAb (Millipore, Billerica, MT, USA).

### Cell growth assay

Cells were seeded in 96-well plastic culture plates (1500/well), in the presence of the indicated drugs, inhibitors or vehicle (DMSO). Cells were fixed in 11% glutaraldehyde and stained with crystal violet at the indicated days after seeding. The dye retained by the cells was then solubilized in 10% acetic acid and the Optical Density (570nm) was measured using a Multilabel Reader (PerkinElmer, Waltham, MT, USA).

### KRAS Fluorescence *in situ* hybridization

FFPE tissue sections were incubated at 56°C for 2-4 h, dewaxed in CitriSolv (Fisher) for 5 min three times, and dehydrated. Tissue permeabilization and protein digestion were carried out by incubations in 2x SSC at 75°C for 15-40 min and in Proteinase K solution (0.6mg/ml in 2X SSC) at 45°C for 20-48 min, respectively. After ethanol dehydration, the probe was applied to the selected hybridization areas, to which a coverslip was added and sealed with rubber cement. DNA co-denaturation was performed at 85°C for 15 min in dry oven. Hybridization was allowed to occur at 37°C for 16 h in humid chamber. Post-hybridization washes were performed by sequential 2-min incubations in 2×SSC/0.3% NP-40 at 74°C and 2×SSC at room temperature. Chromatin was counterstained with DAPI diluted in anti-fade reagent.

Analysis was performed on an epifluorescence microscope using single interference filters sets for green (FITC), red (Texas red), blue (DAPI), gold, aqua, dual (red/green), and triple (blue, red, green) band pass filters. For each interference filter, monochromatic images were acquired and merged using CytoVision (Leica Microsystems Inc). A minimum of 50 tumor nuclei are evaluated per specimen.

To determine *KRAS* gene amplification, the LSI KRAS SpectrumGold and CEP12 (D12Z3) SpectrumGreen probes (Abbott Molecular) were used. The classification was based primarily on the ratio between mean copy number of the gene of interest and the mean copy number of the control probe (CEP12). The specimen was classified as positive for gene amplification when the gene/control ratio was ≥2.0.

### Silencing

The siRNA targeting reagents were purchased from Dharmacon (Lafayette, CO, USA), as a SMARTpool of four distinct siRNA species targeting different sequences of the target transcript. As negative controls, scramble siRNAs that did not target KRAS and NRAS were designed using the GenScript web tool. These were purchased from Sigma-Aldrich and used as a pool. Silencing of KRAS and NRAS expression was achieved by reverse transfection of HCC78 cells with 20 nM siRNAs using Lipofectamine 2000 (Invitrogen, Carlsbad, CA, USA).

### Lentiviral and retroviral transduction

HCC78 cells (1 × 10^5^ cells/35mm plate) were stably transduced with lentiviral/retroviral particles containing constructs encoding wt-KRAS, G12C-KRAS or Q61K-NRAS. As a control, HCC78 cells were infected with lentiviral particles containing the empty vector, pRRL.sin.cPPT.CMV.Wpre. G12C-KRAS was obtained by mutagenesis of wt-KRAS using the QuikChange Lightning Site-Directed Mutagenesis Kit (Agilent Technologies, Santa Clara, CA, USA). Q61K-NRAS was purchased from ADDGENE (Cambridge, MA, USA). KRAS and NRAS mRNA expression levels of infected cells were evaluated 72 hours after cell infection.

### Statistical analyses

Statistical analysis was performed using two-tailed Student's *t* tests. A *P* value of <0.05 was considered significant.

## SUPPLEMENTARY FIGURES AND TABLES


