# Supplementary material for: Activation of RAS family members confers resistance to ROS1 targeting drugs
Source: Oncotarget. 2014 Dec 31;6(7):5182–94. doi: 10.18632/oncotarget.3311 (PMC4467141; doi:10.18632/oncotarget.3311)
Supplement: Supplementary file 1 [file oncotarget-06-5182-s001.pdf]

Activation of RAS family members confers resistance to ROS1 targeting drugs

Supplementary Material

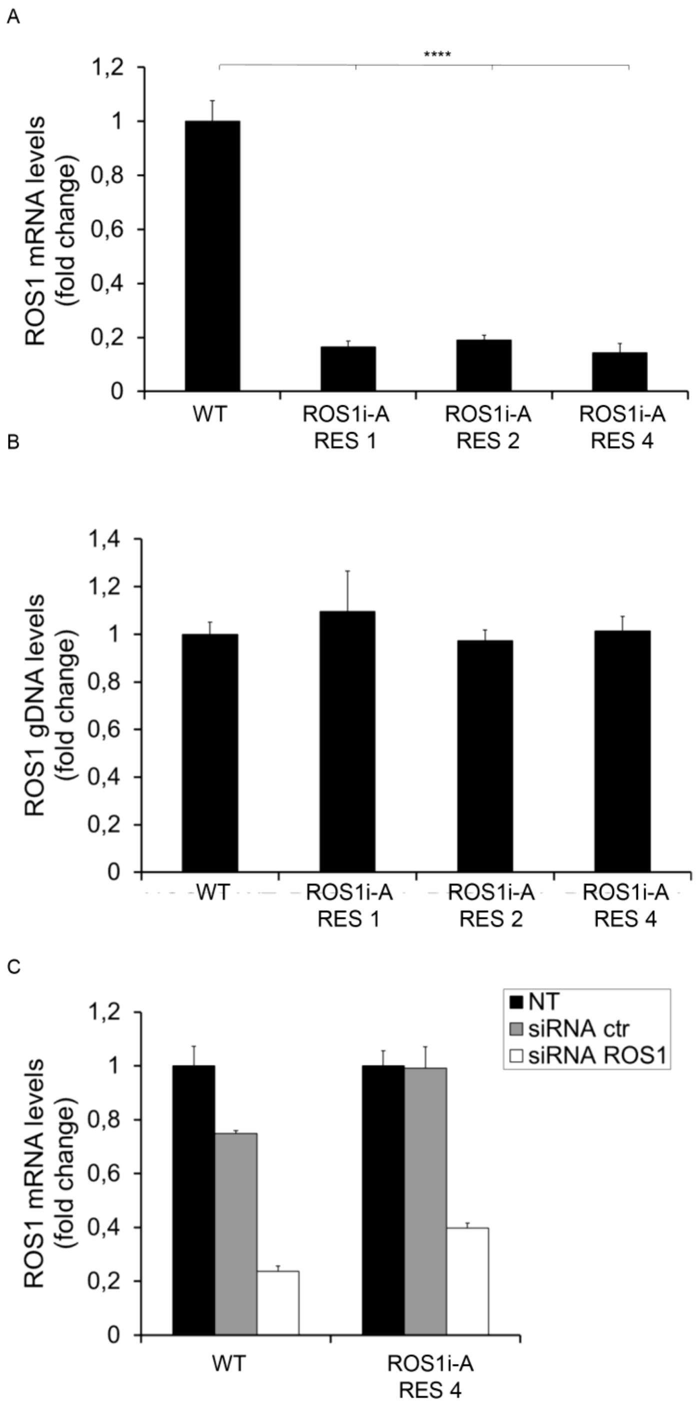

Supplementary FIG. 1: ROS1 expression is decreased in JNJ-ROS1i-A resistant cells, in the absence of gene loss.

A: ROS1 mRNA levels in HCC78 resistant to JNJ-ROS1i-A, evaluated by qRT PCR. The average levels of ROS1 were calculated as fold change difference between the expression in resistant and WT cells. Beta-actin was used as an endogenous control. Error bars represent SEM; ****significantly different from control ($p < 0.0001$). **B:** ROS1 gDNA levels in HCC78 cells resistant to JNJ-ROS1i-A. The ROS1 gene copy number was calculated as fold-change difference between resistant and WT cells. STS6 was used as an endogenous control. Error bars represent SEM. **C:** ROS1 mRNA levels in HCC78 cells transfected with either ROS1 siRNA (siRNA ROS1) or control siRNA (siRNA ctr). The average levels of ROS1 mRNA were calculated as fold-change difference between non transfected (NT) and transfected cells. Beta-actin was used as an endogenous control. Error bars represent SD.

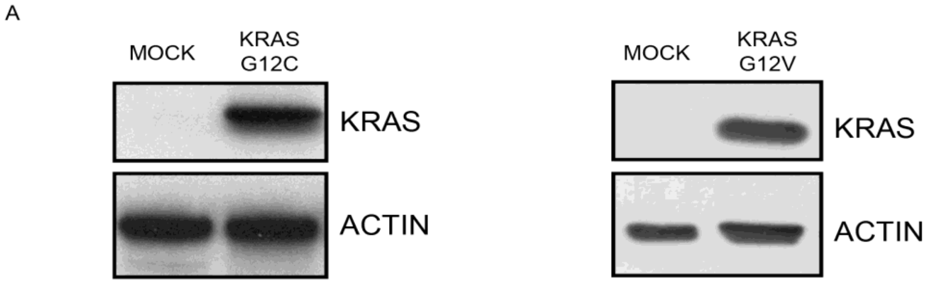

Supplementary FIG. 2: Expression of mutated KRAS in transduced HCC78 cells.

Western blot analysis of KRAS expression in HCC78 cells transduced with either KRAS G12C (left panel) or G12V (right panel) or a MOCK vector. Actin was used as a loading control.

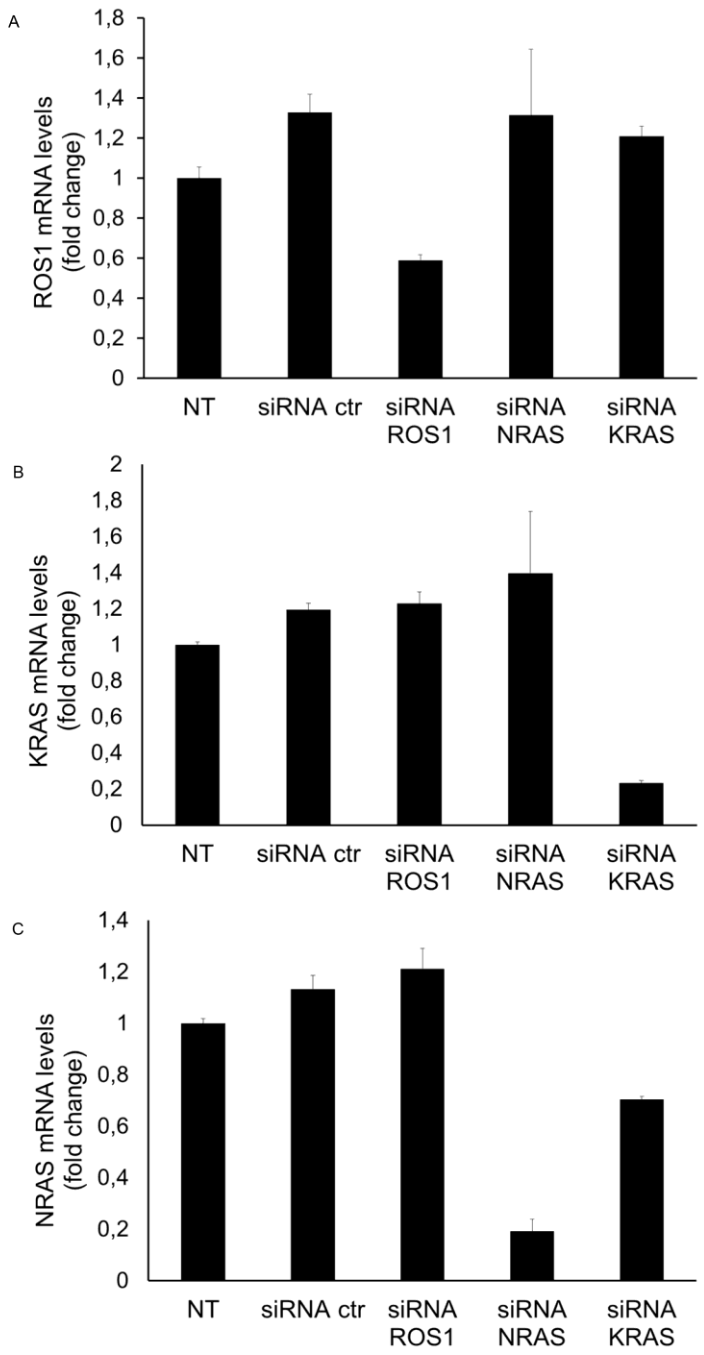

Supplementary FIG. 3: Expression of ROS1, KRAS and NRAS in ROS1i-A-RES 4 cells transfected with different siRNAs.

ROS1i-A-RES 4 cells were transduced with the indicated siRNAs and the expression levels of ROS1 (A), KRAS (B) and NRAS (C) were evaluated by qRT-PCR.

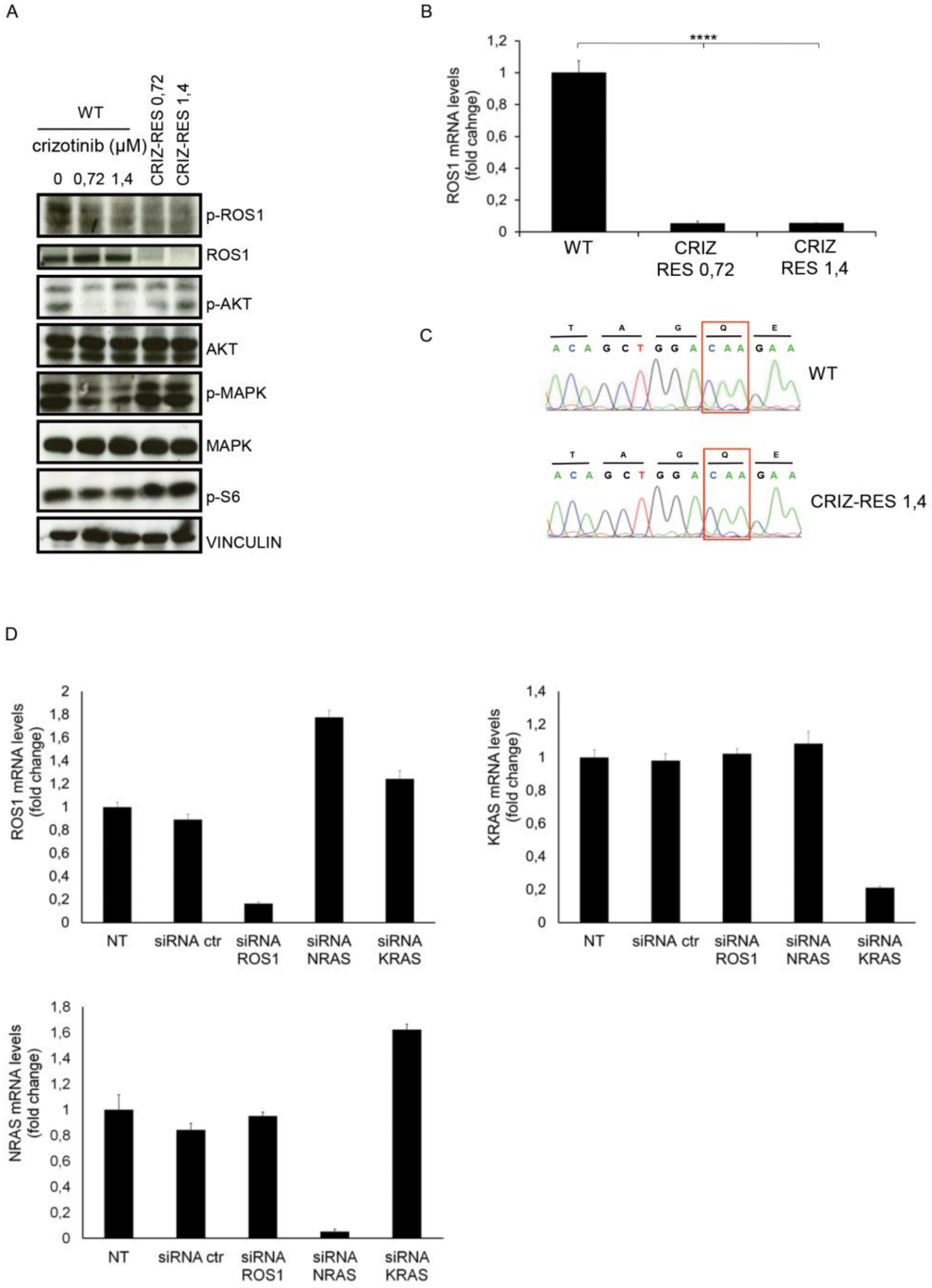

Supplementary FIG. 4; Biochemical and genetic analysis of HCC78 cells resistant to crizotinib.

A: Western blot analysis of HCC78 WT cells (untreated or treated for 4 hours with 0.72 or 1.4 µM crizotinib) or cells resistant to different crizotinib concentrations (CRIZ-RES 0.72 and CRIZ-RES 1.4). The blot was probed with the indicated antibodies. Vinculin has been used as a loading control. **B:** ROS1 mRNA levels in HCC78 cells resistant to crizotinib. The average ROS1 mRNA levels were calculated as fold-change difference between WT and resistant cells. Beta-actin was

used as endogenous control. Error bars represent SD. ****: significantly different from control ($p < 0.0001$). **C:** Sequence analysis of parental (upper panel) and crizotinib resistant cells (lower panel). KRAS codon 12, shown in red, is in the wt configuration in both the cell lines. **D:** CRIZ-RES 1.4 cells were transfected with the indicated siRNAs and the expression levels of ROS1 (left upper panel), KRAS (right upper panel) and NRAS (left lower panel) were evaluated by qRT-PCR.

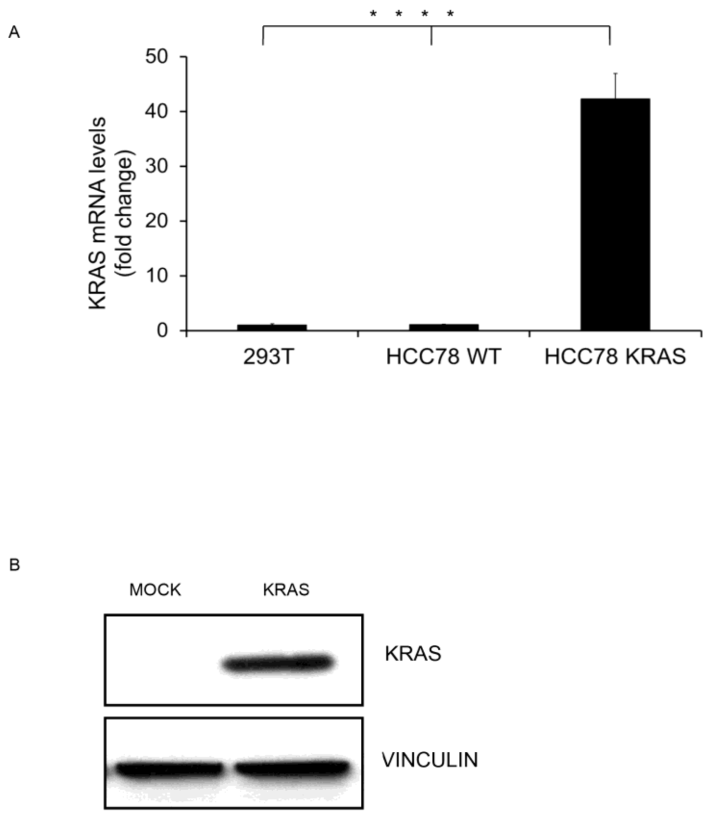

Supplementary FIG. 5: Expression of KRAS in transduced HCC78 cells.

A: KRAS mRNA levels in 293T cells (negative control) and non-transduced HCC78 cells or HCC78 cells transduced with a KRAS expression construct. The average levels of ROS1 mRNA were calculated as fold-change difference between 293T and HCC78 cells. Beta actin was used as an endogenous control. Error bars represent SD. ****: significantly different from control ($p < 0.0001$). **B:** Western blot analysis of KRAS expression in HCC78 cells transduced with either a MOCK vector or a KRAS expression construct. Vinculin was used as loading control.

Supplementary Table 1: Mutational analysis of HCC78 cells resistant to either JNJ-ROS1i-A or crizotinib.

gene	Ros1-A-RES 4		CRIZ-RES 1,4	
	CODON	MUTATION	CODON	MUTATION
KRAS	12	G→C	12	NO
	13	NO	13	NO
	146	NO	146	NO
NRAS	12	NO	12	NO
	13	NO	13	NO
	61	NO	61	Q→K
BRAF	600	NO	NO	NO
PIK3CA	542	NO	NO	NO
	545	NO	NO	NO
	546	NO	NO	NO
	1043	NO	NO	NO
	1047	NO	NO	NO
	1049	NO	NO	NO

Supplementary Table 2: Sequencing primers

gene	exon	primer sequence	
KRAS	1	FW	5' GGTGGAGTATTTGATAGTGTATTAACC
		REW	5' AGAATGGTCCTGCACCAGTAA
	4	FW	5' TGGACAGGTTTTGAAAGATATTTG
		REW	5' ATTAAGAAGCAATGCCCTCTCAAG
NRAS	1	FW	5' GTACTGTAGATGTGGCTCGC
		REW	5' AGAGACAGGATCAGGTCAGC
	3	FW	5' GCAGAAATGGGCTTGAATA
		REW	5' GATTCAGAACACAAAGATCATCC
BRAF	15	FW	5' TGCTTGCTCTGATAGGAAAATG
		REW	5' AGCATCTCAGGGCCAAAAAT
PIK3CA	9	FW	5' GGGAAAAATATGACAAAGAAAGC
		REW	5' CTGCTTTATTTATTCCAATAGGTATGG
	20	FW	5' CTCAATGATGCTTGGCTCTG
		REW	5' TGGAATCCAGAGTGAGCTTTC

Supplementary Table 3: Molecular analysis of re-biopsy samples of evaluable patients

Patient #	Age	Sex	Smoking Status (py)	Histology	Mechanisms of Resistance	
					KRAS, NRAS, BRAF	KRAS FISH
1	38	M	0	Large cell	NA	FOCAL
2	65	M	0	Adenocarcinoma	WT[a]	Not amplified
3	37	F	15	Adenocarcinoma	WT[a]	Not amplified
4	41	M	0	Adenocarcinoma	Failure	Not amplified

[a] SNaPshot® analysis

NA: insufficient
